# Advancing glenohumeral dysplasia treatment in brachial plexus birth injury: the end-to-side spinal accessory to suprascapular nerve transfer technique

**DOI:** 10.1007/s00381-023-06270-y

**Published:** 2024-02-14

**Authors:** Md Sibat Noor, Nathan Khabyeh-Hasbani, Mandana Behbahani, Steven M. Koehler

**Affiliations:** 1 Department of Orthopaedic Surgery, Montefiore Medical Center, Bronx, NY USA; 2 Department of Neurosurgery, Montefiore Medical Center, Bronx, NY USA

**Keywords:** Brachial plexus birth injury, Glenohumeral dysplasia, Nerve transfer, Spinal accessory nerve, Suprascapular nerve

## Abstract

**Purpose:**

Brachial plexus birth injury (BPBI) is a common injury with the spectrum of disease prognosis ranging from spontaneous recovery to lifelong debilitating disability. A common sequela of BPBI is glenohumeral dysplasia (GHD) which, if not addressed early on, can lead to shoulder dysfunction as the child matures. However, there are no clear criteria for when to employ various surgical procedures for the correction of GHD.

**Methods:**

We describe our approach to correcting GDH in infants with BPBIs using a reverse end-to-side (ETS) transfer from the spinal accessory to the suprascapular nerve. This technique is employed in infants that present with GHD with poor external rotation (ER) function who would not necessitate a complete end-to-end transfer and are still too young for a tendon transfer. In this study, we present our outcomes in seven patients.

**Results:**

At presentation, all patients had persistent weakness of the upper trunk and functional limitations of the shoulder. Point-of-care ultrasounds confirmed GHD in each case. Five patients were male, and two patients were female, with a mean age of 3.3 months age (4 days–7 months) at presentation. Surgery was performed on average at 5.8 months of age (3–8.6 months). All seven patients treated with a reverse ETS approach had full recovery of ER according to active movement scores at the latest follow-up. Additionally, ultrasounds at the latest follow-up showed a complete resolution of GHD.

**Conclusion:**

In infants with BPBI and evidence of GHD with poor ER, end-to-end nerve transfers, which initially downgrade function, or tendon transfers, that are not age-appropriate for the patient, are not recommended. Instead, we report seven successful cases of infants who underwent ETS spinal accessory to suprascapular nerve transfer for the treatment of GHD following BPBI.

## Introduction

Brachial plexus birth injury (BPBI) is a relatively common condition, occurring in an estimated 0.5 to 4.6 cases per 1000 live births [1, 2]. A significant portion of affected children, approximately 8–36%, do not fully recover and experience permanent functional impairments [2–5]. The most prevalent consequence is dysfunction of the shoulder [6–8]. As these patients grow older, we observe that they develop shoulders that are internally rotated and have deficits in external rotation, abduction, and forward flexion. They typically have a subluxated shoulder which resembles a posterior dislocation due to retroversion of the glenoid, caused by incomplete innervation of the rotator cuff and deltoid muscles. The progressive shoulder pathology, capsuloligamentous contractures, retroversion of the glenoid, formation of a pseudo-glenoid, and posterior subluxation of the humeral head collectively fall under the term “glenohumeral dysplasia” (GHD) [9].

GHD can have a drastic impact on the quality of life due to the musculoskeletal changes limiting limb function. Hence early diagnosis and intervention are instrumental in preventing long-term complications and achieving full recovery [10]. Confirmatory diagnosis is usually performed with imaging via magnetic resonance imaging (MRI) or ultrasound. MRI allows visualization of the non-ossified cartilaginous structures [11], whereas ultrasound is more useful in young patients (as young as one and a half months old) and can illustrate the location of the humeral ossific nucleus relative to the scapular line [12, 13]. In fact, ultrasound has been shown to be non-inferior to MRI and is now the imaging modality of choice at many centers, including our own [14].

While surgery is the mainstay of treatment, patients often do not necessarily fit clear indications for certain procedures. For example, in an infant with GHD and no external rotation (ER), there is a clear role for performing a spinal accessory to supraspinatus end-to-end nerve transfer [15]. Likewise, while there is literature to support tendon transfers in the child under the age of two [16], the general consensus is that tendon transfers are reserved for toddlers with GHD, as ER tendon transfers have been shown to remodel dysplasia with dwindling efficacy up to age five [17]. However in infants with evidence of GHD and with weak, but present ER, surgeons are faced with a dilemma: whether to downgrade the infant to no ER following an end-to-end nerve transfer in the hopes of achieving a better recovery, or to postpone surgery until the patient is older and suitable for tendon transfers. Moreover, from the perspective of the patient and their family, the psychological impact of downgrading their shoulder function through an end-to-end nerve transfer can be particularly distressing.

Herein, we present our novel technique of reverse end-to-side nerve transfer of the spinal accessory nerve to the suprascapular nerve for correction of glenohumeral dysplasia in brachial plexus birth injuries. Importantly, this technique proves particularly beneficial for patients who fall into the gray area; in which these patients have brachial plexus birth injuries with some, but not functional, external rotation and are too young for tendon transfers. Additionally, we summarize the cases of seven patients who presented with GHD and weakened ER who had successful remodeling of the GHD and a notable improvement in ER strength following this approach, as evidenced by ultrasound findings and assessments of active movement scores.

## Pathoanatomy

**Fig. 1:**
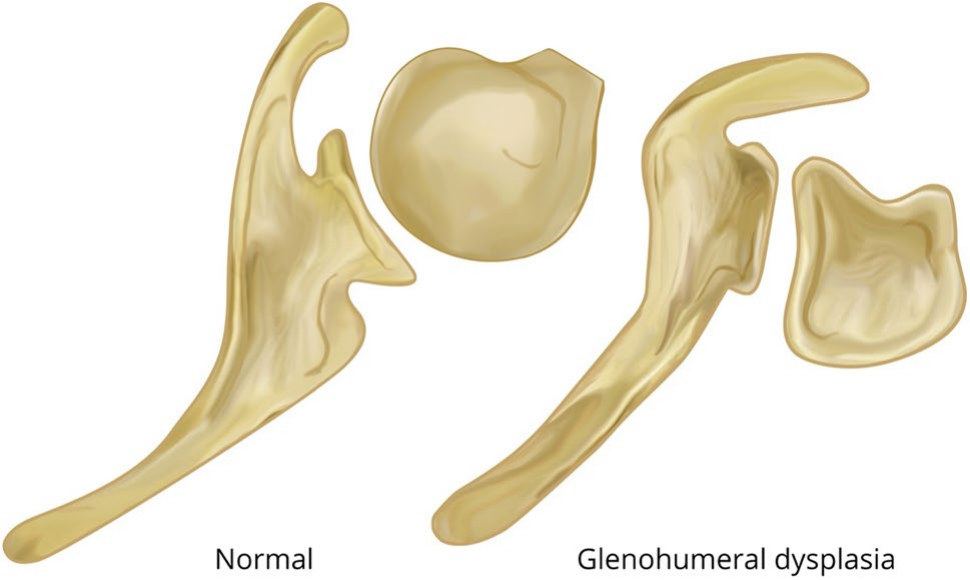
Axial view of changes relating to glenohumeral dysplasia associated with brachial plexus birth injury

**Fig. 2:**
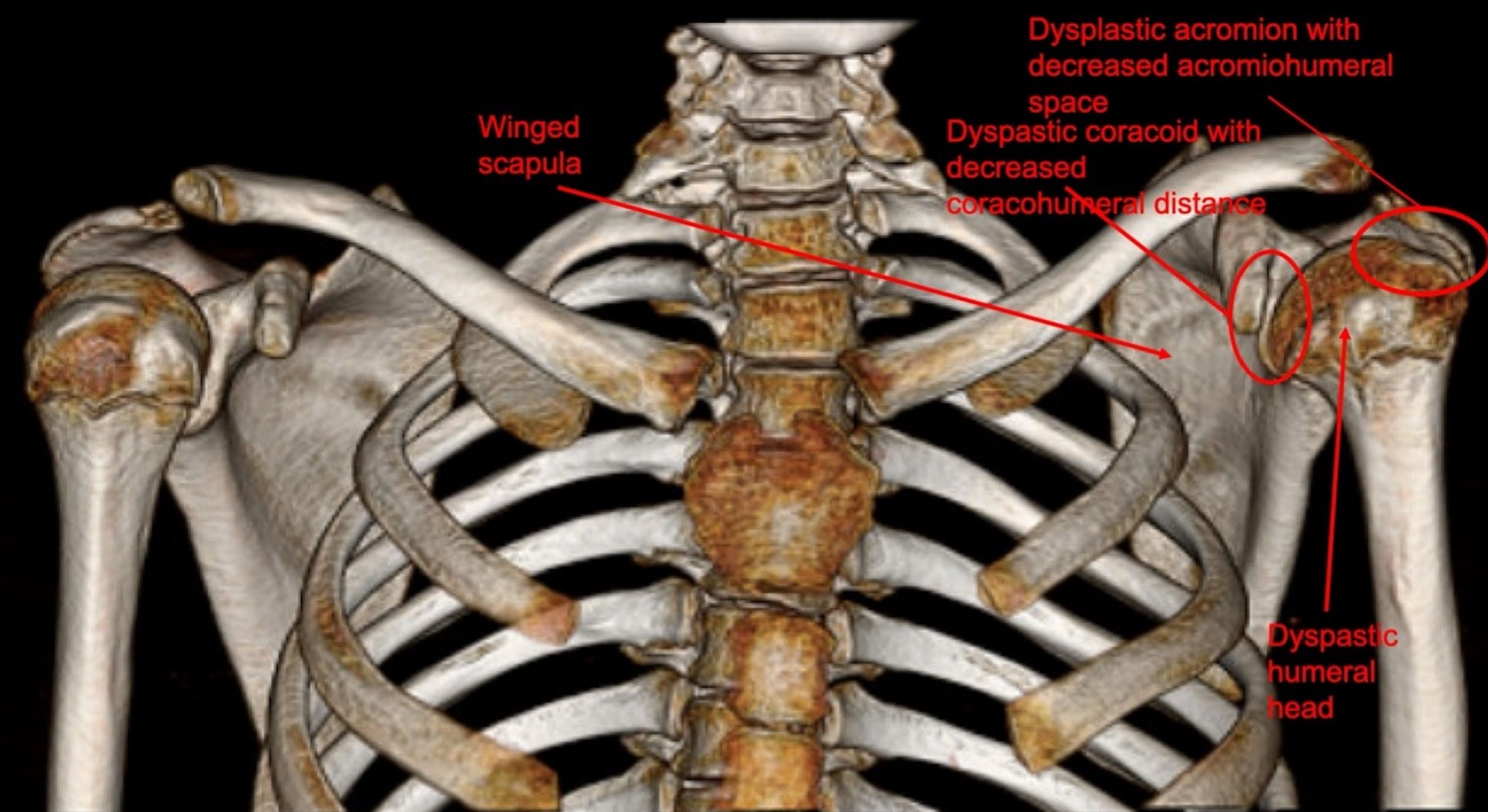
Results of glenohumeral dysplasia that causes the humeral head to become encapsulated in a “cage”

Pearl and Edgerton [18] were the first to describe the stages of glenoid dysplasia, which was later correlated with passive external rotation by Kon et al. [19]. Brachial plexus birth injuries involving the upper roots weaken the supraspinatus, infraspinatus, and rhomboid muscles leading to weakness of external rotation and abduction and causing internal rotation contracture due to unopposed action of the subscapularis and other internal rotators. Over time, this imbalance leads to progressive flattening and retroversion of the humeral head, altering the shape of the glenoid. The glenoid, encompassed by hyaline cartilage, adopts a bi-concave shape, with a false posterior-inferior facet [20]. Gradually, the humerus subluxates posteriorly causing a pseud-glenoid, in which the humeral head articulates with the joint capsule overlying cortical bone (Fig. 1) [7]. Over time, the changes produced limit external rotation resulting in the humeral head becoming encapsulated in a “cage” (Fig. 2).

## Methods

### Exposure

A transverse approach to the supraclavicular plexus along the natural Langer’s line skin folds of the neck is utilized. The platysma is divided, maintaining the lateral third of the sternocleidomastoid. The external jugular vein is identified and retracted, along with the supraclavicular cutaneous nerves which are dissected and retracted. The supraclavicular fat pad is mobilized and retracted superiorly and laterally pedicled off the transverse cervical artery to be utilized at the end of the case for coverage over the plexus. A myotomy is performed on the omohyoid muscle. The internal jugular vein is retracted and the upper trunk of the brachial plexus, the suprascapular nerve, and long thoracic nerves are identified. The suprascapular nerve and the long thoracic nerve are confirmed with intraoperative nerve stimulation. We elect to utilize a handheld nerve stimulator as opposed to formal intraoperative neural monitoring, which is another option. We then divide the anterior and middle scalene muscles to fully expose the brachial plexus roots and trunks.

### Neurolysis

**Fig. 3:**
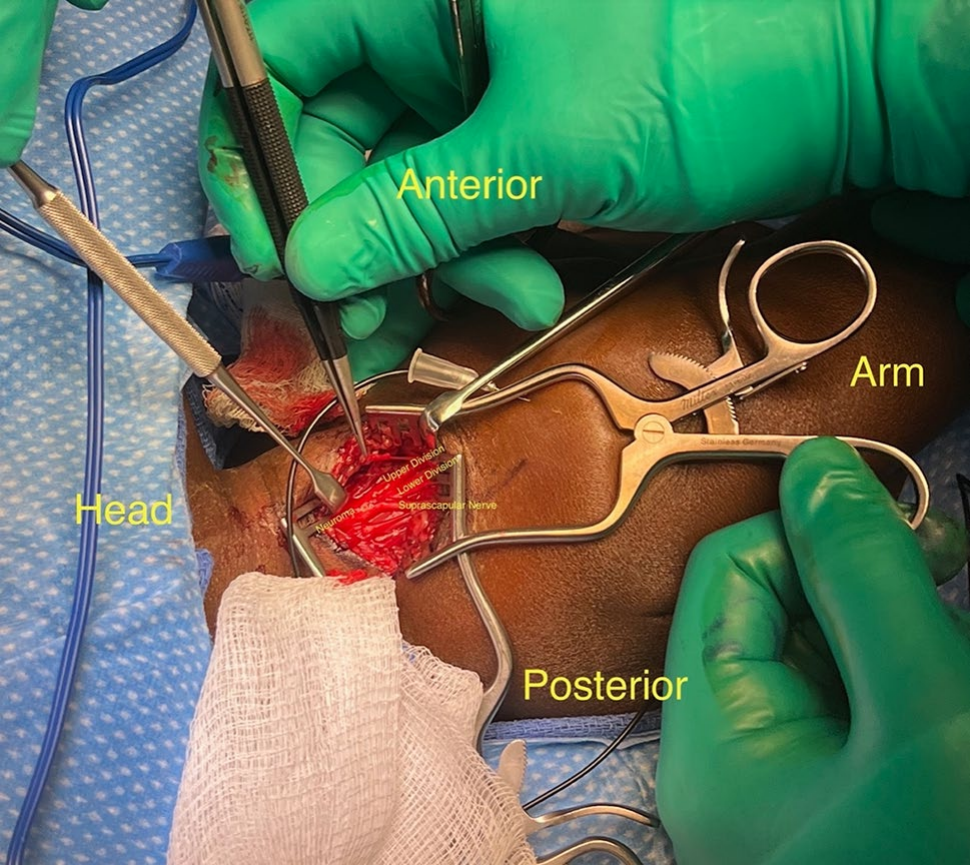
Intraoperative finding of neuroma in situ at C5/6/7 in one patient undergoing reverse end-to-side spinal accessory to suprascapular nerve transfer

In most cases following BPBI, the roots, trunks, and suprascapular nerve are encased in thick cicatrix. In an upper trunk clinical presentation, prior to neurolysis, the C5, C6, and C7 nerve roots are traced to their neuroforamen. In some cases, intraoperative neuromas in situ are discovered (Fig. 3). Nerve avulsions must be excluded.

Conduction prior to neurolysis is recorded. Conduction at 0.5 milliamps (mA) is considered normal if the child demonstrates function under anesthesia that corresponds to an Active Movement Scale (AMS) of 7. Most frequently, we will encounter little to no shoulder ER at 0.5 mA. At 2 mA there may be some supraspinatus function, but often little to no infraspinatus function (AMS of 1 or 2).

Neurolysis of the brachial plexus then commences with systematic removal of the cicatrix from the roots, trunks, divisions, and supraclavicular branches (suprascapular nerve, dorsal scapular nerve, etc.). Repeat nerve stimulation is performed.

### Decision-making

**Fig. 4:**
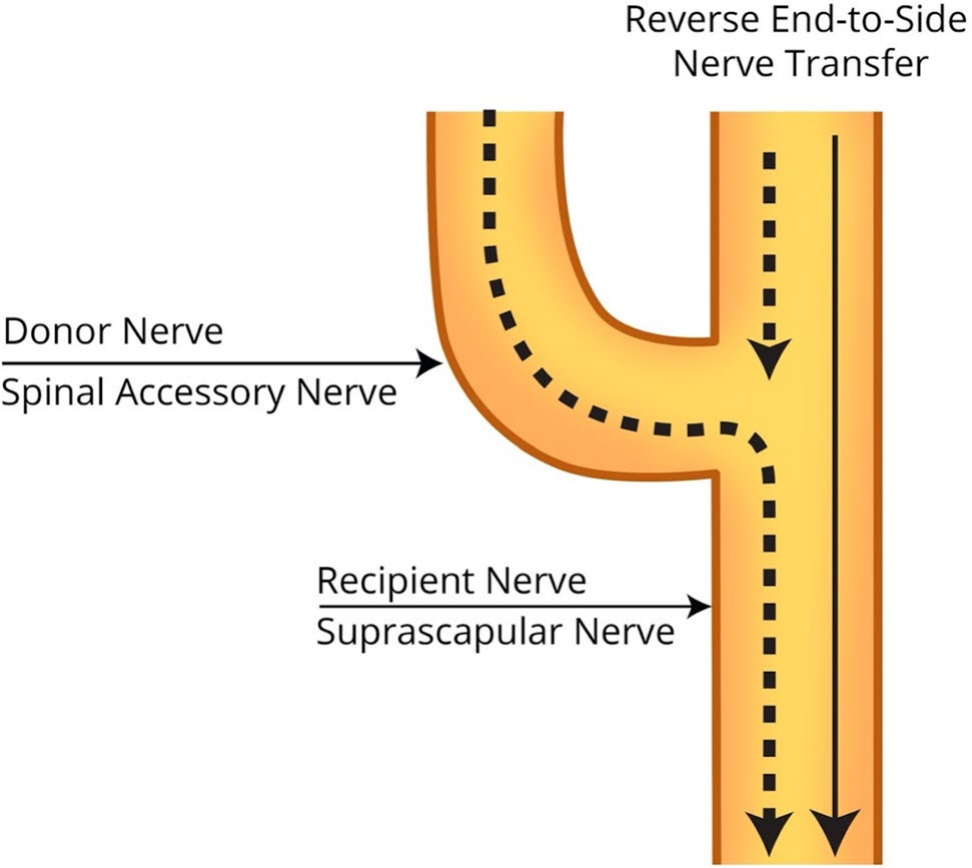
Schematic demonstrating reverse end-to-side nerve transfer of the spinal accessory to the suprascapular nerve

**Fig. 5:**
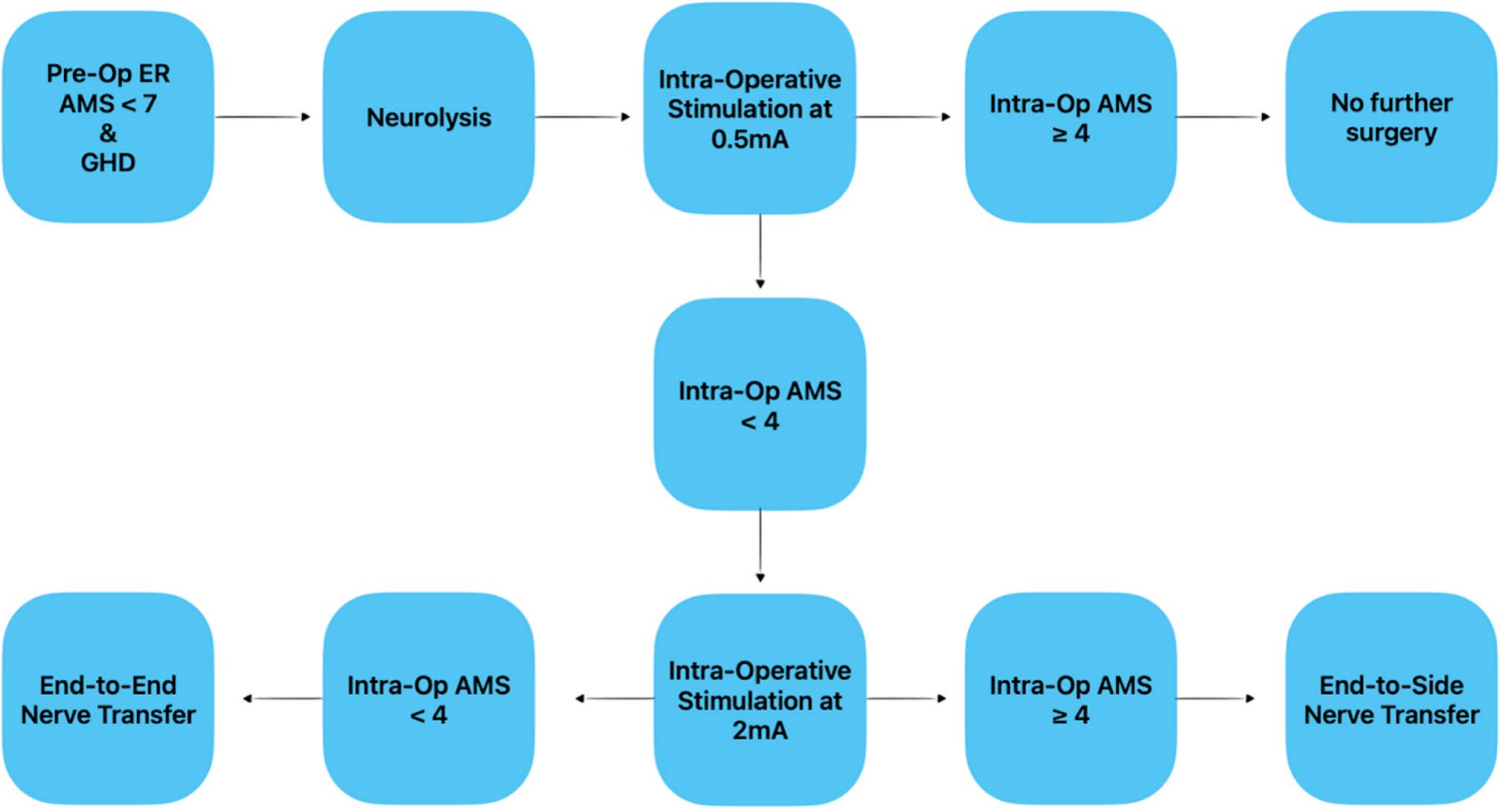
Decision-making diagram for surgical correction of GHD

If intraoperative nerve stimulation at 0.5 mA demonstrates AMS scores of 4 or greater, no further surgery is performed — it is presumed that the child will recover function. If AMS scores of 4 or higher are only achieved with stimulation at 2 mA or greater, then a reverse ETS [21] of the suprascapular nerve with the spinal accessory nerve is performed (Fig. 4). To proceed with an end-to-end nerve transfer, one of two conditions must be met. First, if no conduction is achieved after neurolysis at 2 mA, an end-to-end nerve transfer is performed. Second, if the patient has pre-operative ER AMS scores less than 4 after neurolysis and has intraoperative AMS scores of 3 or less following neurolysis at 2 mA, then an end-to-end nerve transfer is performed (Fig. 5).

### End-to-side technique

**Fig. 6:**
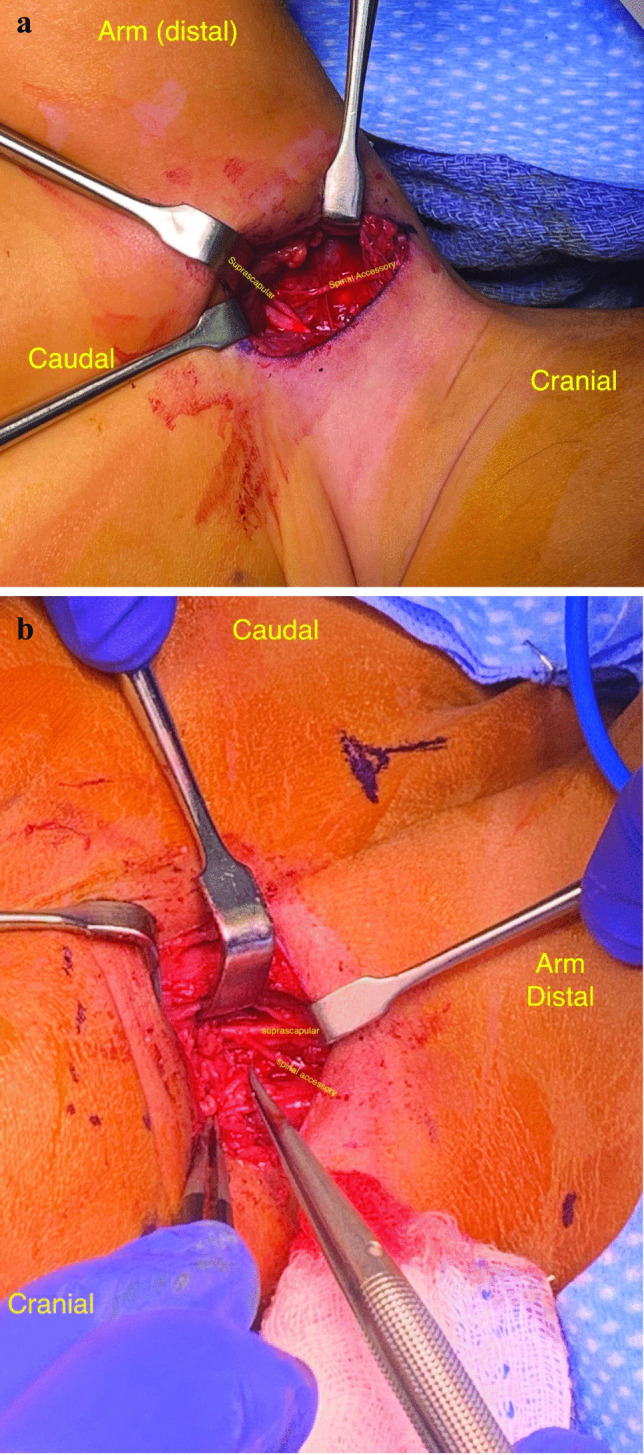
**a, b** Intraoperative images of reverse end-to-side spinal accessory to suprascapular nerve transfer in two patients with glenohumeral dysplasia following brachial plexus birth injury

In the supine position, we utilize Bertelli’s technique for performing the spinal accessory to suprascapular nerve transfer [22]. Micro scissors were then used to open the perineurium of the suprascapular nerve. Next, nerve coaptation using a 9–0 nylon suture of the spinal accessory nerve to the suprascapular nerve is performed (Fig. 6a and b). This is reinforced with fibrin glue and the previously dissected adipofascial flap is mobilized and secured over the plexus.

### Closure and rehabilitation

After surgery, patients are placed in a swathe for 1 week, followed by diligent resumption of brachial plexus therapy.

## Expected results

We performed a reverse (supercharge) end-to-side spinal accessory nerve to suprascapular nerve transfer for the treatment of GHD in seven patients. At the onset of their presentation, all patients’ guardian(s) gave their informed consent for inclusion before they participated in the study. The study was conducted in accordance with the Declaration of Helsinki, and the protocol was approved from the hospital institutional review board. Demographic data, outcome scores, and ultrasound images at the time of diagnosis, as well as post-surgical information from the latest follow-up, were extracted from the patient’s medical records. Operative reports were reviewed for surgical information such as surgical approach, findings, additional nerve transfers or grafting, and any encountered complications.

**Fig. 7:**
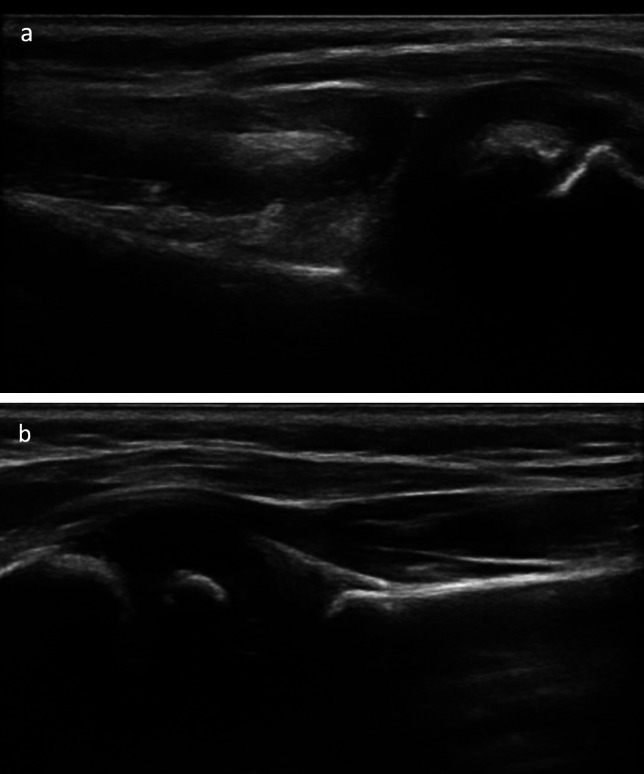
**a, b** Preoperative ultrasounds of two patients demonstrating dysplastic, ossific nucleus posterior to the scapular line indicative of glenohumeral dysplasia following a brachial plexus birth injury

The inclusion criteria comprised of patients who were diagnosed with glenohumeral dysplasia subsequent to BPBIs (Narakas 1–4) [23], and who underwent spinal accessory nerve reverse end-to-side transfer to the suprascapular nerve from the start of 2022 until submission of this manuscript. All patients exhibited persistent weakness of the upper trunk and functional limitations of the shoulder. They were all evaluated with point-of-care ultrasounds depicting the ossific nucleus posterior to the dorsal scapular line indicative of glenohumeral dysplasia **(**see Fig. 7a, b for examples). Among the patients, five patients were male, and two patients were female, with an average age of 3.3 months age (ranging from 4 days to 7 months) at the time of presentation. Surgery was performed on average at 5.8 months of age (3 – 8.6 months). Indications for surgery were based on the surgeons’ expertise and supplemented by relevant literature [24]. Summarized demographic information can be found in Table 1.

**Table 1:** Demographic information of patients who underwent reverse end-to-side spinal accessory to suprascapular nerve transfer

Patient	Sex	Age at presentation	Laterality	Age at surgery	Interval (months)	Age at last follow-up
1	Male	7 months	Right	8.6 months	1.6 months	17 months
2	Male	5 months	Right	5.9 months	0.9 months	12 months
3	Female	4 months	Left	5.7 months	1.7 months	11 months
4	Female	3 months	Right	7.9 months	4.9 months	12 months
5	Male	2 months	Left	3.7 months	1.7 months	8 months
6	Male	4 days	Left	6.5 months	6.5 months	10 months
7	Male	2 months	Left	4.5 months	2.5 months	7 months

All patients underwent preoperative and latest follow-up assessments using the active movement scale (AMS). The AMS scores were used in this study because they demonstrate the best interobserver reliability among current functional outcome measures for children with BPBI [25]. As in other studies [26], we focused on specific AMS sub-scores for shoulder abduction (SA), forward flexion (FF), and external rotation (ER) to evaluate shoulder function. These combined movements synergistically allow the shoulder to operate in multiple planes of motion and rotation [27]. Functional recovery was defined as an AMS score of 6 or higher, with full recovery defined as an AMS score of 7 [28].

**Fig. 8:**
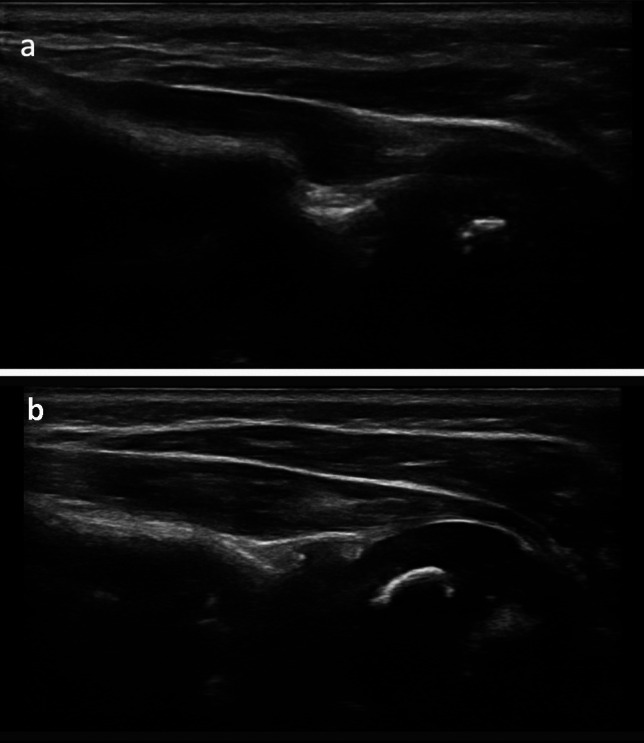
**a, b** Postoperative ultrasounds of two patients from the study which demonstrate an ossific nucleus anterior to the scapular line, indicating a reversal of the glenohumeral dysplasia

The median and range of preoperative AMS scores for SA, FF, and ER were 5 (0–5), 5 (0–5), and 4 (0–5), respectively. In all seven patients treated with a reverse ETS approach, full recovery of ER was achieved, and all patients attained functional recovery of SA and FF shoulder function. The full results are presented in Table 2. Additionally, at each physician visit, patients underwent an ultrasound of the glenohumeral joint to monitor improvements in dysplasia. At the latest follow-up visits, all seven patients exhibited full reversal of glenohumeral dysplasia (see Fig. 8a, b for examples). In addition to the AMS scores, ultrasounds preoperatively and postoperatively are an objective method to follow the path towards improvement of glenohumeral dysplasia. Summarized results of ultrasound findings are also presented in Table 2. The data that support the findings of this study are available on request from the corresponding author.

**Table 2:** AMS Scores and ultrasound findings prior to and following end-to-side spinal accessory to suprascapular nerve transfer

		Pt 1	Pt 2	Pt 3	Pt 4	Pt 5	Pt 6	Pt 7
Shoulder Abduction AMS Score	Initial	5/7	3/7	5/7	4/7	5/7	0/7	4/7
	Postoperatively	6/7	7/7	7/7	7/7	7/7	7/7	7/7
Forward Flexion AMS Score	Initial	5/7	5/7	5/7	4/7	5/7	0/7	4/7
	Postoperatively	6/7	7/7	7/7	7/7	7/7	7/7	6/7
Shoulder External Rotation AMS Score	Initial	5/7	2/7	2/7	4/7	7/7	0/7	4/7
	Postoperatively	7/7	7/7	7/7	7/7	7/7	7/7	7/7
Ultrasound findings of GHD	Initial	Yes	Yes	Yes	Yes	Yes	Yes	Yes
	Postoperatively	No	No	No	No	No	No	No

No surgical complications were recorded. In one case, the patient had an anesthesia complication in which the patient became difficult to ventilate and the anesthesia team problem-solved the ventilation issue, having to replace the endotracheal tube.

## Discussion

Microsurgeries of BPBI infants at an early age have been linked with improved outcomes of AMS scores. For example, surgery before the age of 6 months old has shown better supination recovery, shoulder abduction, and shoulder external rotation [29]. Delay in surgery has been correlated with GHD and poor functional outcomes in adults [30].

GHD can have drastic life-long neurological and psychological complications in children. Early physical signs of impaired shoulder abduction and external rotation in BPBI infants require prompt diagnosis via MRI or ultrasound depending on the age of the child [10–12]. We advocate for aggressive screening of GHD via ultrasound starting as early as 1.5 months old, irrespective of neurological recovery. On ultrasound, the dysplastic, ossific nucleus posterior to the scapular line is pathognomonic to a diagnosis of GHD [31]. If ultrasound is normal, even in the setting of abnormal ER on AMS, then we continue screening with monthly ultrasonography. If by 6 months of age the infant has impaired shoulder ER or signs of GHD on ultrasound, then we indicate the infant for a surgical exploration of the brachial plexus. If the infant develops earlier GHD and it worsens as measured on ultrasonography even in the setting of therapy and splinting (utilizing the supination-external rotation (Sup-ER) orthosis), then we indicate the infant for surgical exploration.

The surgical procedure for GHD is dependent on the patient profile. In many cases of BPBI, there may be the presence of a neuroma in situ, as shown above. Studies have shown that patients with greater than 50% conduction across the neuroma during intraoperative testing tend to benefit from neurolysis alone [32]. Conversely, for patients with less than 50% conduction, indicating more severe disease, a nerve transfer is recommended [32]. In one of our cases, this was exemplified in which neurolysis alone would not account for the resolution of glenohumeral dysplasia confirmed on ultrasound. In our study, intraoperative stimulation at 0.5 mA elicited shoulder abduction and flexion, but no external rotation. When patients were stimulated at 2 mA, AMS grades higher than 1 and less than 6 were obtained. Since our patients had some external rotation at the shoulder girdle after neurolysis (AMS grade greater or equal to 4 with 2 mA of stimulation intraoperatively), we concluded that patients would benefit from a reverse (supercharge) end-to-side nerve transfer.

While literature exists documenting successful enhancement of external rotation and shoulder abduction in BPBI patients through an end-to-end nerve transfer of spinal accessory nerve to suprascapular nerve [33], this involves an initial downgrade in some functions that causes psychological distress for the patient and their families. Additionally, in these children who are still too young for a tendon transfer, but still in the period window in which a nerve transfer is possible, a more logical technique is to not completely conduct a total end-to-end transfer but rather a reverse end-to-side transfer to correct glenohumeral dysplasia.

In the postoperative period, our patients continued their therapy under the guidance of a pediatric brachial plexus therapist. We decided to discontinue the use of a Sup-ER orthosis to evaluate whether the nerve transfer alone could account for the resolution of glenohumeral dysplasia. It is worth noting that preoperative use of Sup-ER splints has shown balanced shoulder growth, muscular function, and improved outcomes in patients recovering from BPBI, as well as prevent the development of Erb’s or extended Erb’s palsies [34]. This also optimized the active functional expression of nerve recovery and limited the need for complete nerve reconstruction in BPBI [5]. In our practice, we routinely employ the Sup-ER splint; however, unlike published data, we do have a rate of continued glenohumeral joint dysplasia necessitating surgery.

Hence, we are reporting a novel procedure for the treatment of GHD following BPBI that has not been published. Given the incidence of BPBI and the drastic long-term effects from BPBI, having a precise treatment protocol can dramatically improve the lives of these patients. Since both tendon transfers as well as end-to-end nerve transfers come with their subset of own limitations, and because not all patients meet the clear-cut criteria for these definitive surgeries, we believe that tackling this gray area with reverse ETS surgery offers the best outcome for these patients. Our results show that following a reverse end-to-side transfer full recovery of shoulder external rotation was obtained and resolution of GHD was demonstrated via ultrasound.

## Limitations

This study has some limitations. First, due to the specific inclusion criteria, our cohort contains a small number of patients. Second, the short follow-up period prevents us from drawing conclusions regarding long-term outcomes. Additionally, we lack a comparative group for result comparison. Although our follow-up data is limited for critical evaluation of the effectiveness of operative intervention, we believe that an end-to-side transfer of the seven cases and ultrasound findings showing beneficial outcomes is the rational approach for treatment in these patients. Further studies with a larger population of patients, and a longer follow-up time are needed to evaluate the long-term effects of end-to-side nerve transfer for the correction of GHD. Additionally, other studies should be conducted to compare the effectiveness of end-to-end nerve transfers and end-to-side nerve transfers for the correction of brachial plexus birth injuries.

## Conclusion

One of the major consequences of brachial plexus birth injuries is shoulder deformity in the form of progressive GHD as the child matures. Effectively managing GHD arising from BPBI requires a nuanced approach, particularly in infants where conventional procedures may not be appropriate. We are the first to report and advocate for an end-to-side spinal accessory to suprascapular nerve transfer for the treatment of GHD following BPBI. This novel technique is designated for patients with evidence of GHD and some external rotation who are still in the window period for a nerve transfer and are not old enough for a tendon transfer. Our successful outcomes of this procedure, resulting in full recovery of external rotation and reversal of glenohumeral dysplasia, in our cohort of patient, offer a valuable addition to the therapeutic arsenal for correcting glenohumeral dysplasia in infants following BPBI.

## Data Availability

Data which supports the reported findings of this study can be retrieved by the corresponding author.
